# A Bibliometric Analysis of the Spatial Transcriptomics Literature from 2006 to 2023

**DOI:** 10.1007/s10571-024-01484-3

**Published:** 2024-06-10

**Authors:** Shu-Han Zhao, Xin-Yu Ji, Guo-Zhen Yuan, Tao Cheng, Hai-Yi Liang, Si-Qi Liu, Fu-Yi Yang, Yang Tang, Shuai Shi

**Affiliations:** 1 Guang’an Men Hospital, China Academy of Chinese Medical Sciences, No. 5 Beixiange Street, Xicheng District, Beijing, 100053 People’s Republic of China; 2 Beijing University of Chinese Medicine, No. 11, Beisanhuan East Road, Chaoyang District, Beijing, 100029 People’s Republic of China; 3 Institute of Basic Research in Clinical Medicine, China Academy of Chinese Medical Sciences, No. 16 Nanxiaojie, Dongzhimennei Ave, Beijing, 100700 People’s Republic of China; 4 School of Chinese Medicine, Beijing University of Chinese Medicine, No. 11, Beisanhuan East Road, Chaoyang District, Beijing, 100029 People’s Republic of China

**Keywords:** Spatial transcriptomics, Bibliometric analysis, Tissue recognition, Cancer, Differentiation, Models

## Abstract

In recent years, spatial transcriptomics (ST) research has become a popular field of study and has shown great potential in medicine. However, there are few bibliometric analyses in this field. Thus, in this study, we aimed to find and analyze the frontiers and trends of this medical research field based on the available literature. A computerized search was applied to the WoSCC (Web of Science Core Collection) Database for literature published from 2006 to 2023. Complete records of all literature and cited references were extracted and screened. The bibliometric analysis and visualization were performed using CiteSpace, VOSviewer, Bibliometrix R Package software, and Scimago Graphica. A total of 1467 papers and reviews were included. The analysis revealed that the ST publication and citation results have shown a rapid upward trend over the last 3 years. Nature Communications and Nature were the most productive and most co-cited journals, respectively. In the comprehensive global collaborative network, the United States is the country with the most organizations and publications, followed closely by China and the United Kingdom. The author Joakim Lundeberg published the most cited paper, while Patrik L. Ståhl ranked first among co-cited authors. The hot topics in ST are tissue recognition, cancer, heterogeneity, immunotherapy, differentiation, and models. ST technologies have greatly contributed to in-depth research in medical fields such as oncology and neuroscience, opening up new possibilities for the diagnosis and treatment of diseases. Moreover, artificial intelligence and big data drive additional development in ST fields.

**Graphical Abstract:**

Spatial transcriptomics (ST) technologies and application prospects. (1) Imaging-based approaches, including in situ sequencing (ISS)—where transcripts are amplified and sequenced in tissue—and ISH-based approaches—where imaging probes are sequentially hybridized in tissue. (2) NGS-based techniques, in which positional information is encoded onto transcripts prior to NGS sequencing.

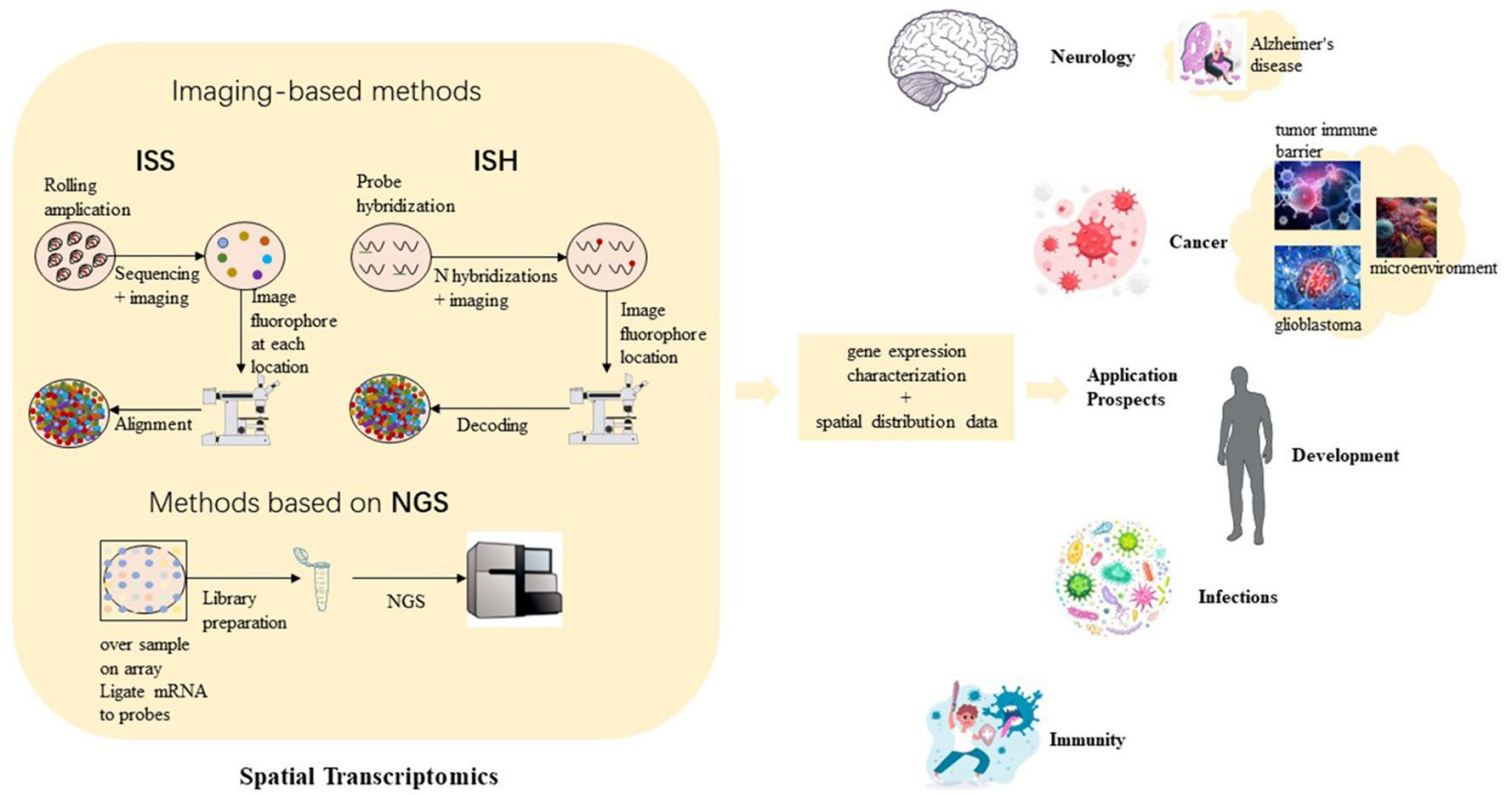

**Supplementary Information:**

The online version contains supplementary material available at 10.1007/s10571-024-01484-3.

## Introduction

Over the last decade, transcriptomics technology has rapidly developed, and if we categorize it at the research level, it has gone through three main stages: bulk transcriptome sequencing, single-cell transcriptome sequencing, and spatial transcriptomics (ST). The main difference between the bulk transcriptome and single-cell transcriptome is the type of information the two approaches generate (Mortazavi et al. 2008). In the analysis of heterogeneous biological systems, the bulk transcriptome only provides information on the average gene expression of the sample, whereas the single-cell transcriptome allows the investigation of transcriptional heterogeneity among individual cells. Spatial transcriptomics provides information on the spatial distribution of gene expression profiles, thereby elucidating interesting features that were not previously revealed by single-cell RNA sequencing methods, which do not provide spatial information (Park et al. 2023). In addition, combining single-cell and ST for joint studies enables gene expression studies at spatial locations to be combined to the single cell level at ultrahigh resolution, which has promising applications in the fields of cancer, immunity, neurology, developmental biology, and so on (Ozato et al. 2023; Valberg et al. 2023).

In biology, the concept of space is very important. To generate organisms, cells undergo a process that involves a complex combination of different information layers to form tissues, which subsequently form organs and systems (Giacomello 2021). Each cell in a multicellular organism interacts with its surroundings (Park et al. 2023), so measuring the location of molecules in a tissue is essential for understanding the formation and function of tissues, considering that the location of each cell in a tissue is often critical to its function (Giacomello 2021). To understand the complexity of biological systems, ranging from various physiological phenomena to the pathology of disease, it is necessary to assess the function of individual cells and their interactions to coordinate the complex functions of tissues and organs (Park et al. 2023). A comprehensive understanding of how individual cells use their mRNAs and proteins in different tissues of the body may lead to new strategies for the prevention or treatment of infections, cancer, neurological or metabolic disorders, and a large number of other diseases (Williams et al. 2022).

The ST refers to techniques used for analyzing the spatial information of transcriptomes. It is a collection of groundbreaking new genomics technologies that measure gene expression and provide information on the spatial localization in tissues. Techniques for counting and analyzing transcripts in tissues have been around for decades, and in 2021, ST was recognized by Nature Methods as “Method of the Year 2020.” (Marx 2021) This further confirms that technology has great development potential that is wide in scope and that subsequent spatial transcriptome technologies will change the way we understand complex biological organization in various research areas. The field continues to evolve rapidly, driven by a number of factors, including reductions in the cost of next-generation sequencing (NGS), programs such as the Human Cell Atlas (HCA) and the Brain Initiative Cellular Census Consortium (BICCC) (Ecker et al. 2017; Regev et al. 2017), increases in computational power, and improvements in microscopy and imaging technologies. Recent technological advances based on next-generation sequencing and imaging methods have established the power of ST to systematically measure the expression levels of all or most genes across an entire tissue space and have been used to reveal biological insights in neuroscience, developmental biology, and plant biology, as well as to study a range of disease contexts, including cancer (Rao et al. 2021). As ST has seen an explosion in the number of studies in recent years, it has become an important frontier technology in the biomedical field and may have a significant impact on cancer, immunity, neurology, and other fields in the future. Through a literature search, the first relevant literature in 2006 explored the transcriptional and metabolic responses of E. coli to dissolved oxygen tension gradients at the spatial level, illustrating the origin of the discipline's development (Lara et al. 2006). In order to see the development of the discipline more comprehensively, we chose to perform a literature visualization of ST from 2006 to 2023.

To identify key issues for future research, bibliometric analysis, the study of scholarly publications, is often used to provide a comprehensive picture of the state of research in a given field (Guler et al. 2016). First introduced in 1969, bibliometric analysis has become a well-developed, interdisciplinary science combining bibliography, statistics, and mathematics and is a comprehensive and effective methodology for analyzing scientific information. Bibliometric analysis quantitatively evaluates and visualizes the contributions of authors, articles, journals, institutions, and countries, as well as the links between them (Brandt et al. 2019; Chen et al. 2012; Wilson et al. 2021). The computational platforms for bibliometric analysis, CiteSpace and VOSviewer software, automatically generate interactive visual networks by analyzing records of scientific publications (Chen et al. 2012). Bibliometric analysis has been widely used in various areas of medical research, including cardiology, oncology, immunology, and public health, due to its powerful predictive capabilities for research prospects (Ge et al. 2022; Meng et al. 2022; Xavier-Santos et al. 2022; Xu et al. 2022a, 2022b; Yang et al. 2022; Zhang et al. 2022).

Based on the bibliometric analysis of publications included in the WoSCC (Web of Science Core Collection) Database, this study aimed to (i) identify the intractable problems and research hotspots related to spatial transcriptomics; (ii) reveal the research trend of spatial transcriptomics over the past decade or so; (iii) construct a knowledge graph for this field; and (iv) provide valuable insights for future related research.

## Methods

### Data Collection

The literature search was conducted in April 8, 2024, using the WoSCC database (https://www.webofscience.com/wos/woscc/basic-search) with a search time limit of January 1, 2006 to December 31, 2023. We used the following strategy: (((TS = (spatial transcriptomics)) OR (TS = (in situ hybridization)) AND ALL = (spatial transcriptomics)) OR (TS = (in situ sequencing)) AND ALL = (spatial transcriptomics)) OR (TS = (Next Generation Sequencing)) AND ALL = (spatial transcriptomics).

We limited the type of literature to articles and reviews. There was no restriction on language. We completed all retrievals and extractions on April 8, 2024, within the same day. The retrieved records were saved as plain text files and named download_txt.

### Data Analysis

Documents exported from the WoSCC Database were imported into CiteSpace 6.3.R1 (Chen 2004), VOSviewer 1.6.18 (van Eck and Waltman 2010), Scimago Graphica (Hassan-Montero et al. 2022), and Bibliometrix R Package software. Cite Space 6.3.R1 was used to analyze keyword cluster analysis and detect emerging keywords. VOSviewer 1.6.18 was used for visual analysis of countries/regions and institutions, author and co-cited author visualization, journal co-citation analysis, keyword collinearity, and density analysis. The visualization of intercountry collaborative networks was generated using Scimago Graphica. Bibliometrix R Package software (Aria and Cuccurullo 2017) is an opensource tool designed by Aria, M and Cuccurullo, C programmed in R for performing comprehensive scientific map analysis. In this study, it helps to show the average annual citation volume and the annual publication volume in this field with the form of line charts and capture the dynamic changes of annual subject terms and journal development.

**Fig. 1:**
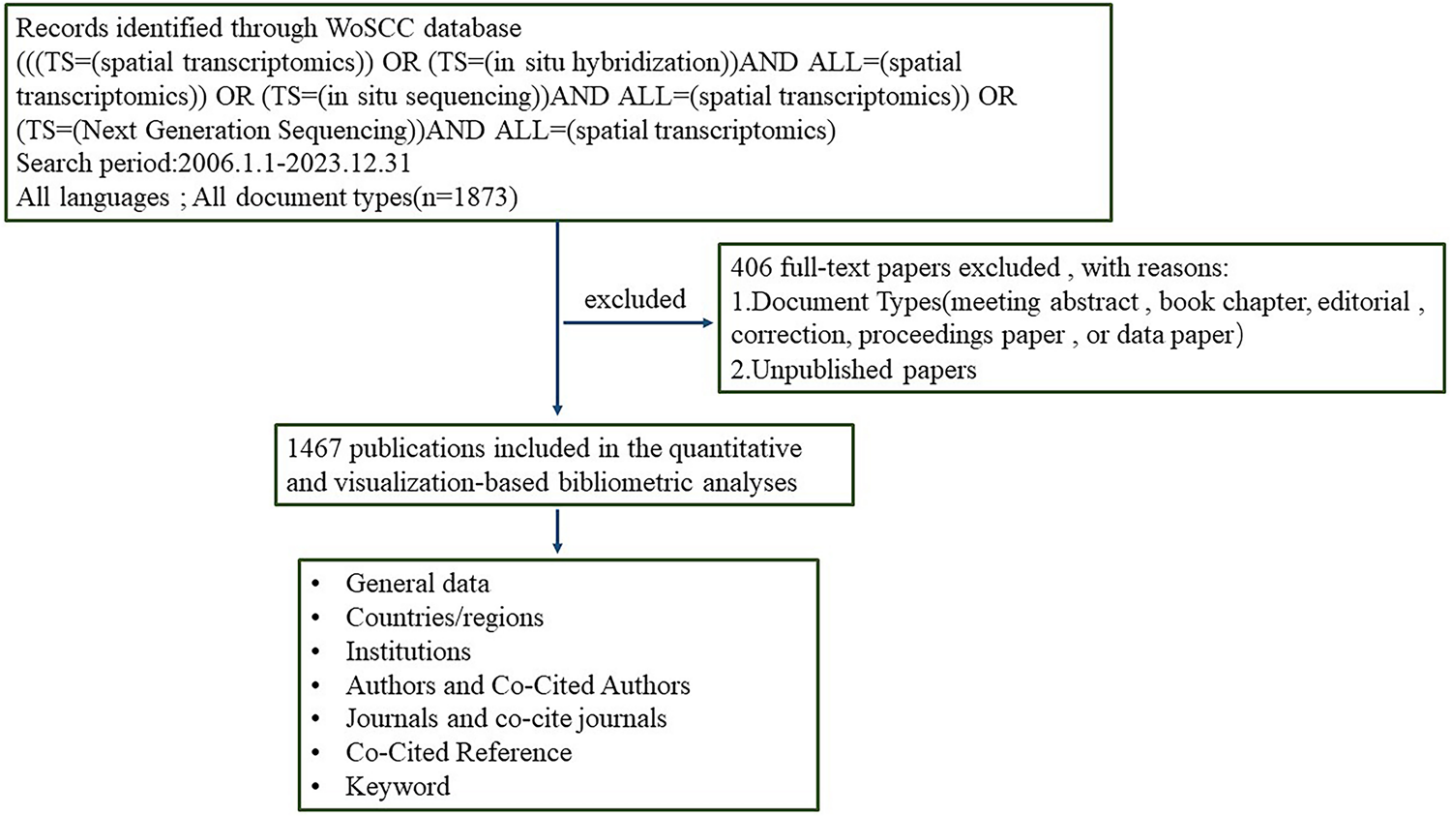
Flowchart for spatial transcriptomics bibliographic analysis. *TS* Topics

The process for literature screening and analysis in this study is shown in Fig. 1.

## Results

### Annual Trends in Publications and Citations

**Fig. 2:**
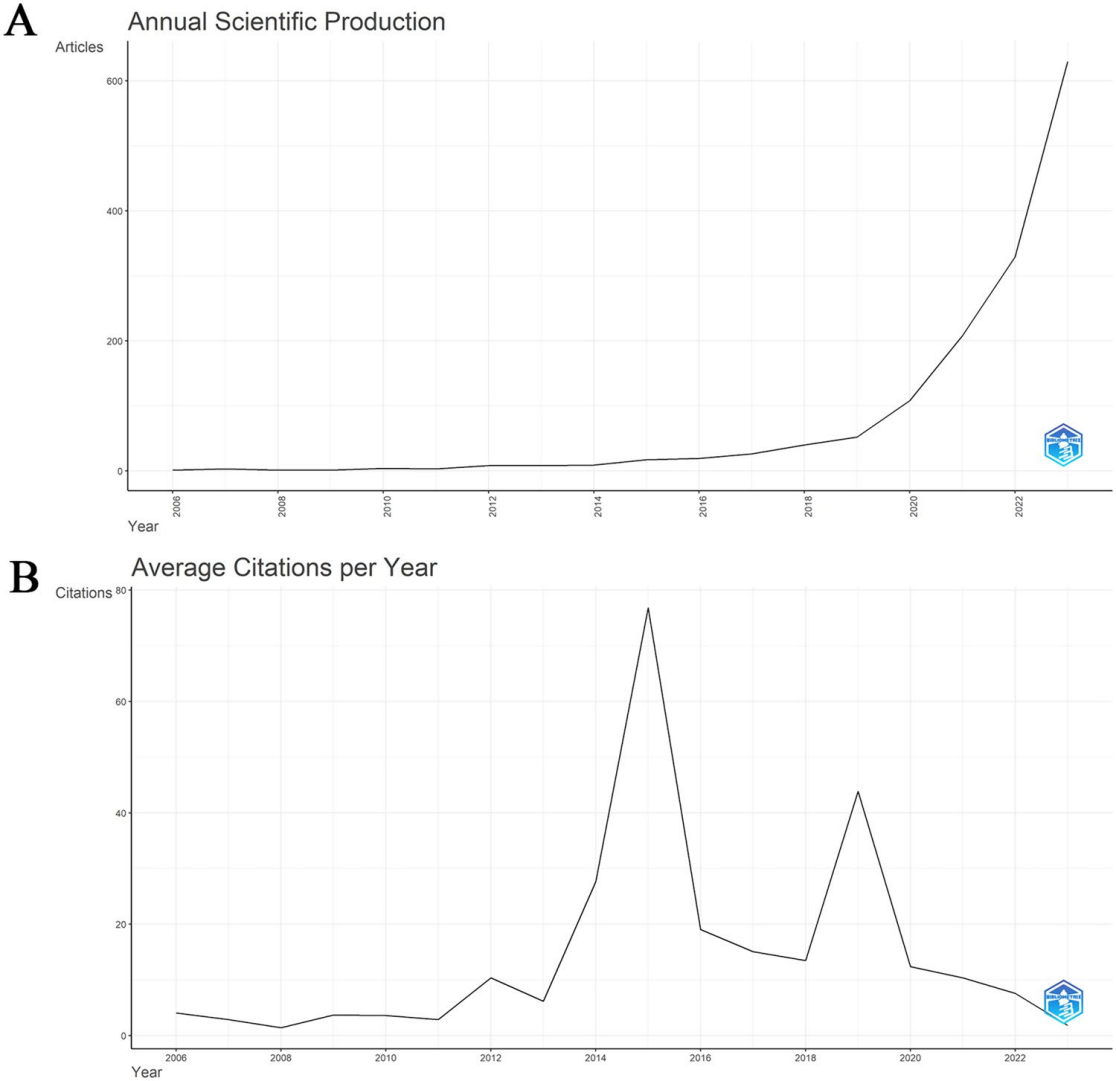
The annual publication and annual average citation trends from 2006 to 2023. **A** The annual publication. **B** The annual average citation trends

According to the search process, a total of 1467 studies published from 2006 to 2023 were included in the bibliometric analysis, as shown in Fig. 2. The total number of citations for the retrieved literature was 50,505, with an average of 41.57 citations. The dynamics of the number of publications over the last decade reflect the general trend in the field. As shown in Fig. 2, the number of studies published from 2006 to 2019 is relatively small, and the publication output, although steadily increasing, has a small annual growth, with an annual number of publications that is still less than 100 by the end of 2019. Beginning in 2020, when spatial transcriptomics was selected as the “Method of the Year 2020” an increasing number of scholars began to focus on this field, and the annual output of publications in this field increased rapidly, reaching the peak at 630 in 2023. Overall, research on spatial transcriptomics is still expected to be in a phase of rapid growth based on publication volume trends and deserves much attention in future research.

### Analysis of Journals and Co-cited Journals

A total of 489 journals published 1467 papers on spatial transcriptomics during the time period studied. The top ten journals are shown in Table 1. Among them, Nature Communications is in first place, with a total of 107 papers published and an impact factor of 16.6, followed by Nature (*n* = 37) and Bioinformatics (*n* = 36), both of which have published 30 spatial transcriptomics papers and which have impact factors of 64.8 and 5.8, respectively. Eight of top 10 journals are in the Q1 JCR division, and 4 journals have an IF of more than 10, with Nature having the highest impact factor of 64.8. Nature has the highest impact factor of 64.8. Seven of the top 10 journals are from the UK, and 2 are from the CH.

**Table 1:** Top 10 journals related to spatial transcriptomics

Rank	Journal	N	Country	IF (2022)^a^	JCR (2022)
1	Nature Communications	107	UK	16.6	Q1
2	Nature	37	UK	64.8	Q1
2	Bioinformatics	36	UK	5.8	Q1
4	Frontiers in Immunology	26	CH	7.3	Q1
5	Nature Biotechnology	26	UK	46.9	Q1
6	Nature Methods	23	UK	48	Q1
7	Scientific Reports	23	UK	4.6	Q2
8	Briefings in Bioinformatics	22	UK	9.5	Q1
9	Cancers	21	CH	5.2	Q2
10	Cell Reports	21	US	8.8	Q1

*UK* The United Kingdom, *US* The United States, *CH* Switzerland

^a^Impact factor 2022 is an indicator extracted from the JCR database (https://jcr.clarivate.com/jcr/browse-journals)

**Fig. 3:**
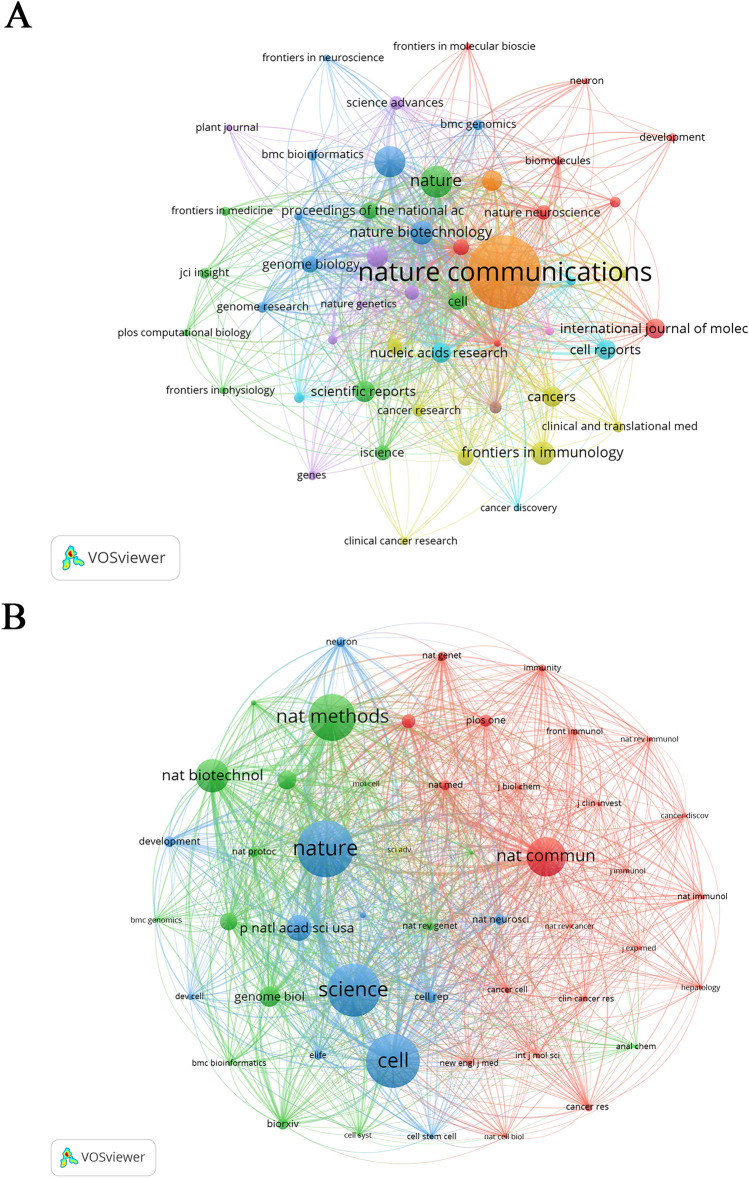
The network visualization maps of **A** citing and **B** co-cited journals

The impact of a journal in a research area depends on the number of citations. The network visualization maps of citing and co-cited journals were performed with VOSviewer (Fig. 3). The size of the nodes represents the number of citations, and the lines between the nodes indicate co-citation relationships. As for the colors of the cluster analysis, in the cluster analysis of citing journals, journals were classified into nine clusters, with yellow mainly related to cancer and immunity, and blue representing the field of genetics and biology. In the cluster analysis of co-cited journals, the blue color mainly represents authoritative academic journals, such as Nature, Cell, and Science; green represents academic journals in the field of gene, biology, and technology research; and red represents academic journals in the field of immunology and cancer. From the journal analysis, we can tentatively conclude that ST technology is inextricably linked to cancer and immunity. A total of 7909 co-cited journals were found for the period of the last 18 years, 13 journals were cited more than 1000 times, and 7 journals were cited more than 2000 times. As shown in Table 2, Nature is the most cited journal with 5020 citations, followed by Cell with 4762 citations. Among the top 10 co-cited journals, Nature had the highest impact factor (64.8), followed by cell (64.5). All 10 citing and co-cited journals belonged to the Q1 region of the JCR, and there were 5 journals with an impact factor of > 40, which demonstrated that the papers were high-quality spatial transcriptomics-related literature and underscores the academic significance of the present study. It can also be found that the highly citing and co-cited journals generally belong to the top journals and their sub-journals, which shows a trend consistent with the academic level of the journal.

**Table 2:** Top 10 co-cited journals related to spatial transcriptomics

Rank	Co-cited journal	Number of Citations	Country	IF (2022)^a^	JCR (2022)
1	Nature	5020	UK	64.8	Q1
2	Cell	4762	US	64.5	Q1
3	Science	4661	US	56.9	Q1
4	Nature Methods	4166	UK	48	Q1
5	Nature Communications	3507	UK	16.6	Q1
6	Nature Biotechnology	2970	UK	46.9	Q1
7	Proceedings of the National Academy of Sciences of the United States of America	2275	US	11.1	Q1
8	Genome Biology	1827	UK	12.3	Q1
9	Nucleic Acids Research	1716	UK	14.9	Q1
10	Bioinformatics	1535	UK	5.8	Q1

*UK* The United Kingdom, *US* The United States

^a^Impact factor 2022 is an indicator extracted from the JCR database (https://jcr.clarivate.com/jcr/browse-journals)

We also visualized the top 50 cited and co-cited journals with a spectral density plot (Supplemental Fig. 1) and found that some journals, e.g., Nature Communications, Nature, and Cell, appeared in both plots, which suggests that these journals maintain a close connection with the spatial transcriptomics research field.

### Analysis of Active Countries/Regions and Institutions

**Fig. 4:**
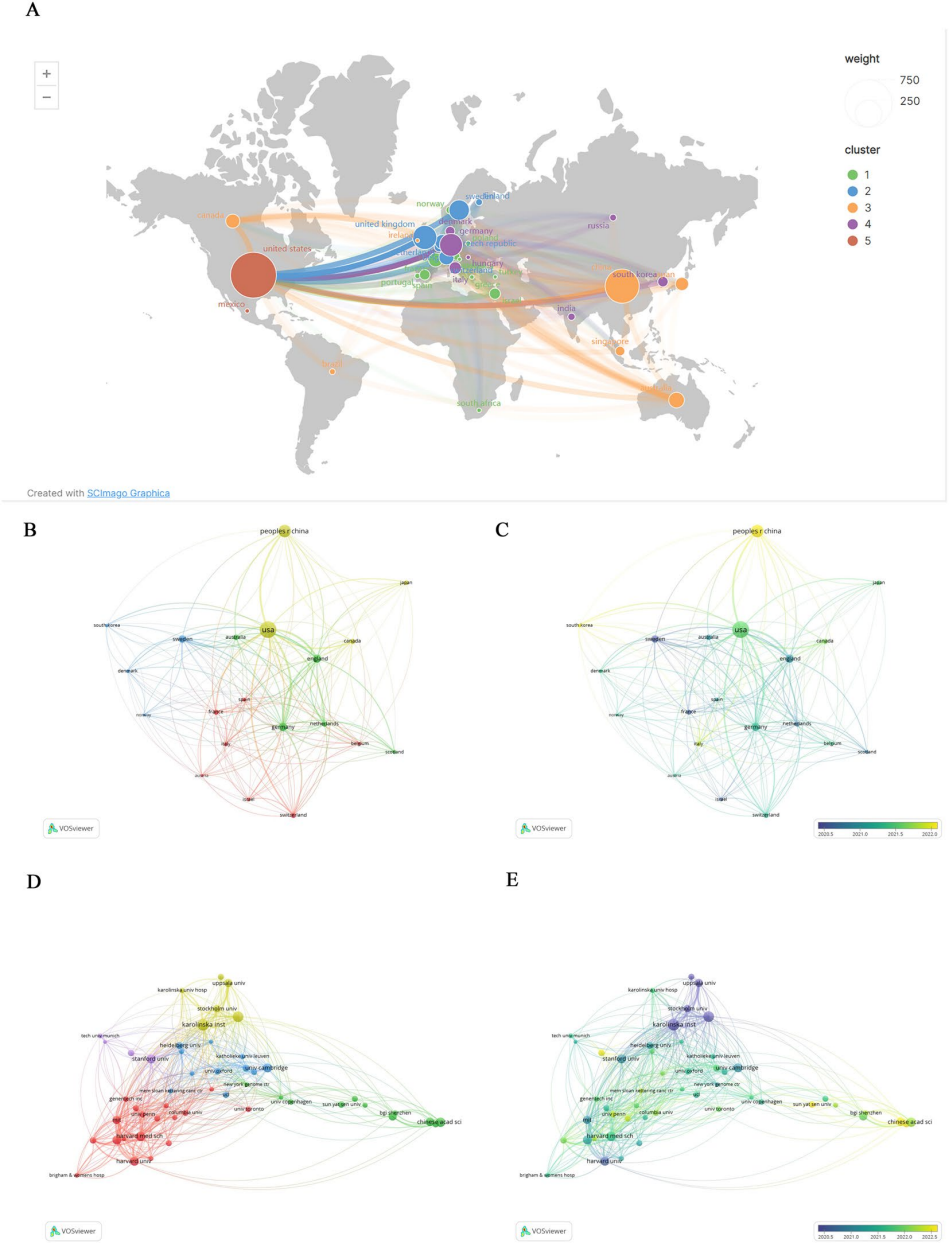
Collaboration networks among countries and among institutions. **A** The collaboration map among countries. **B** The cooperation network among the top 20 most productive countries. **C** The time-overlay map of the cooperation network among the top 20 most productive countries. **D** The cooperation network among the top 50 most productive institutions. **E** The time-overlay map of the cooperation network among the top 50 most productive institutions

A total of 1467 studies were published by 2049 institutions from 65 countries/regions in the last decade. Among these countries, the United States ranked first with 659 global publications, followed by China (*n* = 366), the United Kingdom (*n* = 197), and Germany (*n* = 163) (Table 3). These four countries accounted for more than 80% of global publications. Nevertheless, as of 2022, most countries have fewer than 30 publications, and some regions still are not represented among the publications in this field (Fig. 4A).

**Table 3:** Top 10 countries related to spatial transcriptomics

Rank	Country	Documents	Total citations	Citations per article
1	The United States	659	33,311	50.5477997
2	China	366	5493	15.00819672
3	The United Kingdom	197	10,238	51.96954315
4	Germany	163	14,443	88.60736196
5	Sweden	130	22,750	175
6	Australia	77	2377	30.87012987
7	Switzerland	70	2625	37.5
8	Netherlands	69	4360	63.1884058
9	Canada	62	1150	18.5483871
10	France	61	1778	29.14754098

Interestingly, China has the second highest number of publications but the fifth highest number of citations to papers, whereas the opposite is true for Sweden, which has the second highest number of citations to papers, an average number of literature citations and the fifth highest number of publications. This may be related to limitations in access to the literature and choice of language.

To explore collaboration between countries, we used VOSviewer as well as Scimago Graphica to analyze the data and generate visualization output. As shown in Fig. 4A, the network of collaboration between countries is complex and extensive.

Collaboration centers, indicated by the intersection of the line segments, are mainly located in North America (US), Northern Europe (Sweden), Asia (China), and Western Europe (UK).

The top 20 countries' national collaborative author networks were automatically clustered into three categories, represented by four colors (Fig. 4B). The temporal overlay visualization map showed that production in these countries was concentrated over 2 years (Fig. 4C). Considering these observations as well as the clustering results, it can be assumed that the green clusters are more active than the others, and within each cluster, some members have become more active in the past 1 year, such as South Korea in the blue cluster (Fig. 4B, C).

In total, more than 2,000 institutions conduct research in spatial transcriptomics, and the top 10 most productive institutions, of which 4 are located in the United States and 2 in Sweden, are shown in Table 4. The Karolinska Institute was ranked first with 72 papers, and the top two institutions with the most publications and total citations were all Swedish institutions: KTH Royal Institute of Technology (63/19478) and Karolinska Institute (72/19069). The collaboration of the top 50 research institutions was similarly analyzed by a network of co-authors. A total of five clusters are shown (Fig. 4D), with the largest cluster (red) consisting mainly of USA institutions.

**Table 4:** Top 10 organizations related to spatial transcriptomics

Rank	Organizations (Country)	Counts	Total citations	Citations per article
1	Karolinska Inst (Sweden)	72	19,069	264.8472222
2	Kth Royal Inst Technol (Sweden)	63	19,478	309.1746032
3	Harvard Med Sch (US)	49	4474	91.30612245
4	Chinese Acad Sci (CHN)	46	663	14.41304348
5	Harvard Univ (UK)	44	8342	189.5909091
6	Broad Inst Mit & Harvard (US)	43	8306	193.1627907
7	Stanford Univ (US)	43	3188	74.13953488
8	Univ Cambridge (UK)	43	4424	102.8837209
9	Univ Chinese Acad Sci (CHN)	39	330	8.461538462
10	MIT (US)	37	5855	158.2432432

*UK* The United Kingdom, *US* The United States, *CHN* China

Of these clusters, the yellow cluster was most active during the early 2020s, while the green clusters were more active in the last year of the year, suggesting that Swedish institutions started spatial transcriptomics research earlier but that Chinese institutions such as Chinese Academy of Sciences followed closely behind and generated more new research (Fig. 4E).

### Analysis of Authors and Co-cited Authors

**Fig. 5:**
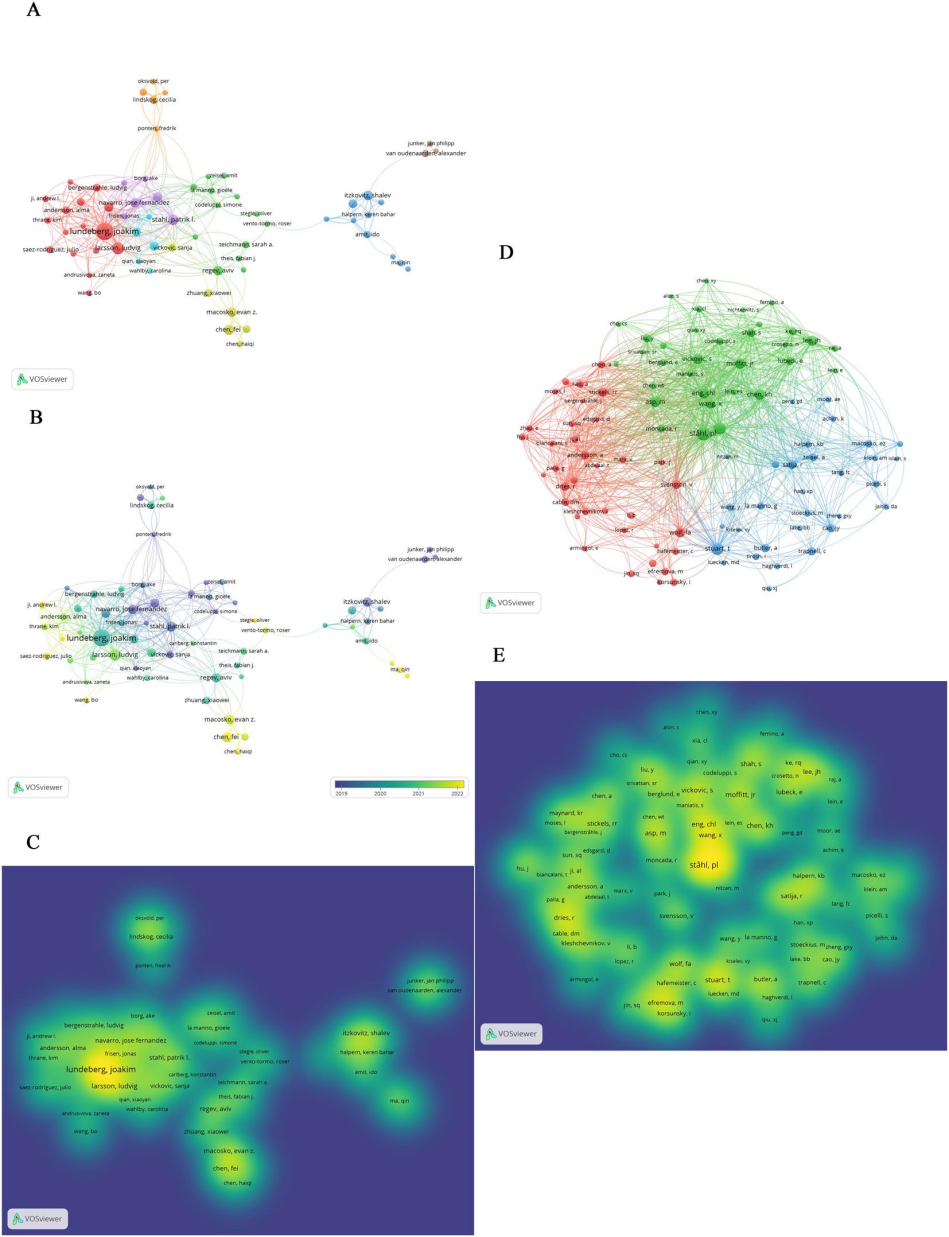
Scholar cooperation maps. **A** The cooperation network among the top 100 most productive authors. **B** Time-overlay maps of the top 100 most productive authors. Each node represents an author, and each line represents the link between two authors. **C** The spectral density map of the top 100 most productive authors. **D** The cooperation network among the top 100 most productive co-cited authors. **E** The spectral density maps of the top 100 most co-cited authors

A total of 10,086 authors have been involved in spatial transcriptomics publications in the last 18 years. The 100 most productive authors and co-cited authors are shown in spectral density plots in Fig. 5C, E. Detailed information on the top 10 authors in both rankings is shown in Table 5. Among the top 10 most productive authors, one of them being from Asia and the rest are being from America and Europe, there are 11–41 published papers and 711–5074 citations per author. Joakim Lundeberg of the Royal Institute of Technology (*n* = 41) has published the most papers, followed by Ludvig Larsson of the Royal Institute of Technology and Stahl, Patrik l. of Royal Institute of Technology, with 19 and 15 publications, respectively. While the total number of citations of the co-cited authors ranged from 20 to 432, 53 were cited more than 100 times, and 10 were cited more than 200 times. The top 5 co-cited authors were Ståhl, Pl (*n* = 432), Stuart, T (*n* = 317), Rodriques, Sg (*n* = 308), Moffitt, Jr (*n* = 253), and Chen, Kh (*n* = 247) (Table 6).

**Table 5:** Top 10 most productive authors related to spatial transcriptomics

Rank	Author	Institution (Country)	Documents	Total citations	Citations per article
1	Lundeberg, Joakim	Kth Royal Inst Technol (Sweden)	41	5074	123.7560976
2	Larsson, Ludvig	Kth Royal Inst Technol (Sweden)	19	1379	72.57894737
3	Stahl, Patrik l	Kth Royal Inst Technol (Sweden)	15	2623	174.8666667
4	Chen, Fei	Blood Ctr Zhejiang Prov (CHN)	14	1001	71.5
5	Macosko, Evan Z	Harvard University (US)	13	1165	89.61538462
6	Itzkovitz, Shalev	Broad Institute (US)	12	1609	134.0833333
7	Navarro, Jose Fernandez	Vall d'Hebron Institut d'Oncologia (VHIO), Royal Institute of Technology (UK)	12	2758	229.8333333
8	Regev, Aviv	Broad Inst Massachusetts Inst Technol MIT & Harvard (US)	12	4279	356.5833333
9	Mollbrink, Annelie	Lund University Faculty of Medicine, Div Gene Technol (Sweden)	11	2202	200.1818182
10	Nilsson, Mats	Kristianstad University (Sweden)	11	711	64.63636364

*UK* The United Kingdom, *US* The United States; CHN, China

**Table 6:** Top 10 co-cited authors related to spatial transcriptomics

Rank	Co-cited authors	Institution (Country)	Total citations	Total link strength
1	Stahl, Patrik L	Kth Royal Inst Technol (Sweden)	432	12,207
2	Stuart, Tim	New York University (US)	317	9085
3	Rodriques, Samuel G	Francis Crick Institute; Appl Biotechnol Lab,1405 Minnesota St (UK)	308	10,948
4	Moffitt, Jeffrey R	Boston Children's Hospital; Program Cellular & Mol Med, Dept Microbiol (US)	253	9141
5	Chen, Kok Hao	A*STAR—Genome Institute of Singapore (Singapore)	247	9285
6	Asp, Michaela	Kth Royal Inst Technol (Sweden)	244	8541
7	Sanja Vickovic	Clin Ctr Vojvodina; Dept Anesthesiol & Intens Care, University of Novi Sad Medical Faculty (Serbia)	235	9249
8	Eng, C.-H. L	California Institute of Technology (US)	233	8826
9	Lee, Jun Hee	Fort Valley State University Georgia Small Ruminant Res & Extens Ctr, Agr Res Stn (US)	227	8437
10	Wolf, Felix A	Swiss Federal Institutes of Technology Domain (Sweden)	205	6768

*UK* The United Kingdom, *US* The United States

Collaborative networks among the top 100 most productive authors and among co-cited authors were categorized into eight and three categories, respectively, based on the closeness of ties (Fig. 5A, D). An overlay visualization combining years (Fig. 5B) shows that the orange, purple, and green clusters were most active in approximately 2019, and red and yellow clusters are more active in the last 2 years, while the brown and blue clusters represent literature published mainly in 2019–2021 and lacked strong connections with the other clusters.

The influence of co-cited authors in the field is shown in the density plot (Fig. 5E). Among the co-cited authors, Patrik L. Ståhl ranks first, and the author with the highest centrality is Rodriques, Sg (0.10) of New York University, indicating that this author dominates the research field.

### Analysis of Co-cited Reference

We analyzed a total of 71,501 references and listed the top 10 in Table 7. The Science article titled Visualization and analysis of gene expression in tissue sections by spatial transcriptomics ranked first with a frequency of 432 citations. The top ten co-authored articles were published in top international journals and their sub-journals such as Science, Cell, Nature, etc., all with more than 100 citations.

**Table 7:** Top 10 co-cited references related to spatial transcriptomics

Rank	Author	Title	Journal	Citation	Year	IF (2022)^a^
1	Stahl, Patrik L	Visualization and analysis of gene expression in tissue sections by spatial transcriptomics	Science	432	2016	56.9
2	Rodriques, Samuel G	Slide-seq: A scalable technology for measuring genome-wide expression at high spatial resolution	Science	302	2019	56.9
3	Stuart, Tim	Comprehensive Integration of Single-Cell Data	Cell	270	2019	64.5
4	Chen, Kok Hao	Spatially resolved, highly multiplexed RNA profiling in single cells	Science	247	2015	56.9
5	Eng, Chee-Huat Linus	Transcriptome-scale super-resolved imaging in tissues by RNA seqFISH	Nature	218	2019	64.8
6	Vickovic, Sanja	High-definition spatial transcriptomics for in situ tissue profiling	Nature Methods	197	2019	48
7	Satija, Rahul	Spatial reconstruction of single-cell gene expression data	Nature Biotechnology	165	2015	46.9
8	Stickels, Robert R	Highly sensitive spatial transcriptomics at near-cellular resolution with Slide-seqV2	Nature Biotechnology	163	2021	46.9
9	Wolf, F. Alexander	SCANPY: large-scale single-cell gene expression data analysis	Genome Biology	163	2018	12.3
10	Wang, Xiao	Three-dimensional intact-tissue sequencing of single-cell transcriptional states	Science	159	2018	56.9

^a^Impact factor 2022 is an indicator extracted from the JCR database (https://jcr.clarivate.com/jcr/browse-journals)

### Keyword Analysis

**Fig. 6:**
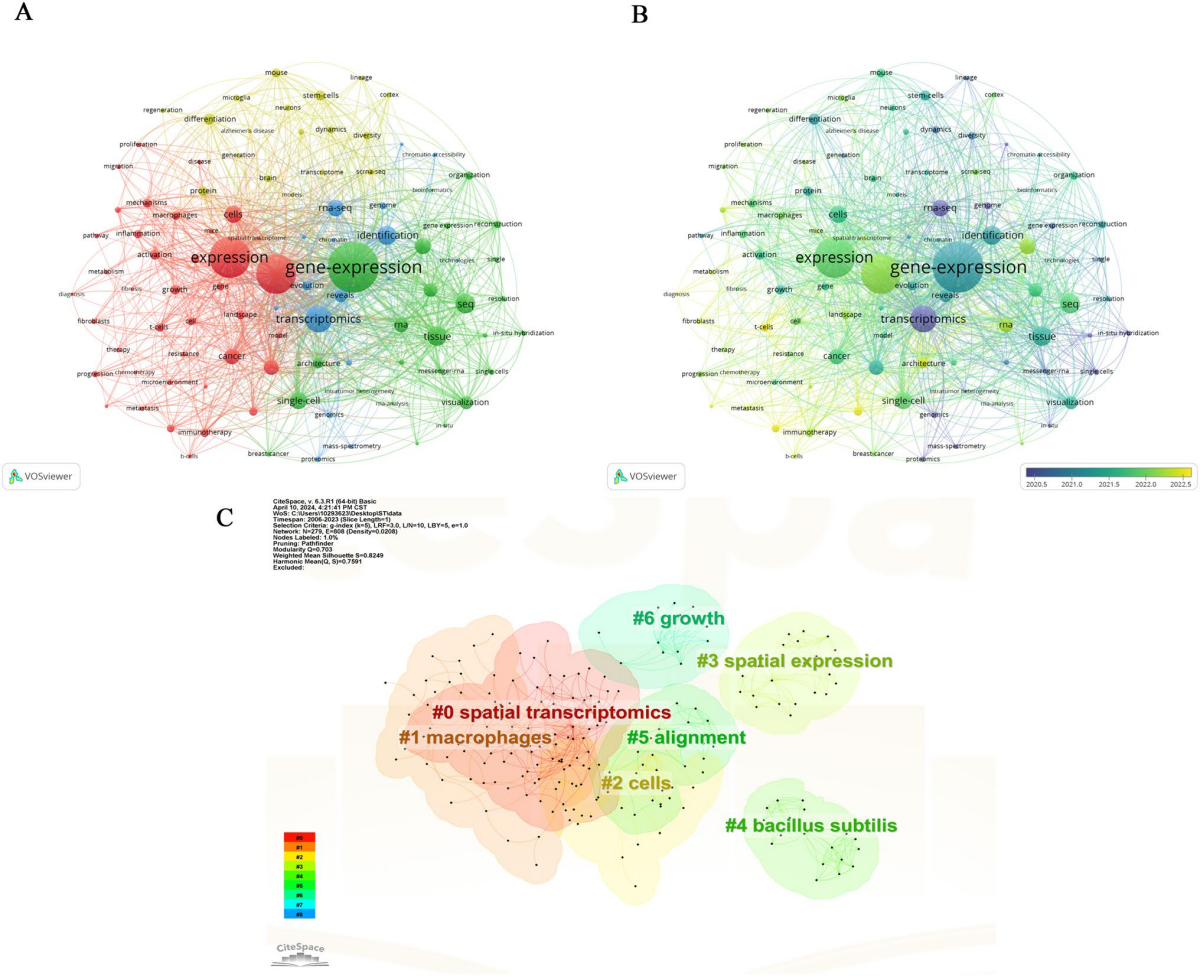
Knowledge map of keywords. **A** The cooperation network among the top 100 keywords. **B** Time-overlay maps of the top 100 keywords. **C** Cluster diagrams of keywords. **D** The spectral density maps of the top 100 keywords

Keyword analysis can reflect the current status of research topics in ST fields, including research hotspots and future directions. We used VOSviewer to construct high-frequency keyword co-occurrence networks and visualize them (Fig. 6A). Table 8 shows the top 25 keyword co-occurrence terms.

**Table 8:** Top 25 co-cited keywords related to spatial transcriptomics

Rank	Keyword	Total link strength	Occurrences
1	Gene expression	1501	410
2	Expression	861	313
3	Spatial transcriptomics	1141	304
4	Transcriptomics	612	185
5	Seq	572	126
6	Tissue	564	126
7	Identification	424	118
8	Cells	310	106
9	Single-cell	452	104
10	RNA-seq	358	100
11	Atlas	406	96
12	Cancer	363	92
13	Heterogeneity	402	87
14	RNA	330	84
15	Genome-wide expression	393	80
16	Reveals	312	75
17	Visualization	392	75
18	Differentiation	203	62
19	Activation	183	56
21	Stem cells	189	56
22	Architecture	247	55
23	Protein	169	52
24	Evolution	196	50
25	Growth	166	49

The keyword “gene expression” has the highest number of mentions at 410 and the highest total link strength at 1501. Combined with a visual overlay for the years of interest (Fig. 6B), its color is cyan, indicating that the average publication year is the first half of 2021. The terms “expression,” “genome-wide expression,” and “gene expression” are the most frequently mentioned, similar to “gene-expression,” with 313 and 33 occurrences, and their total link strengths are 861 and 114, respectively. Throughout the study period, we used “single-cell,” “single-cell rna sequencing,” “scrna-seq,” “cell rna-seq,” “rna-seq data,” “single-cell sequencing,” and other keywords related to cell sequencing, with the results indicating that cell sequencing is closely related to ST.

**Fig. 7:**
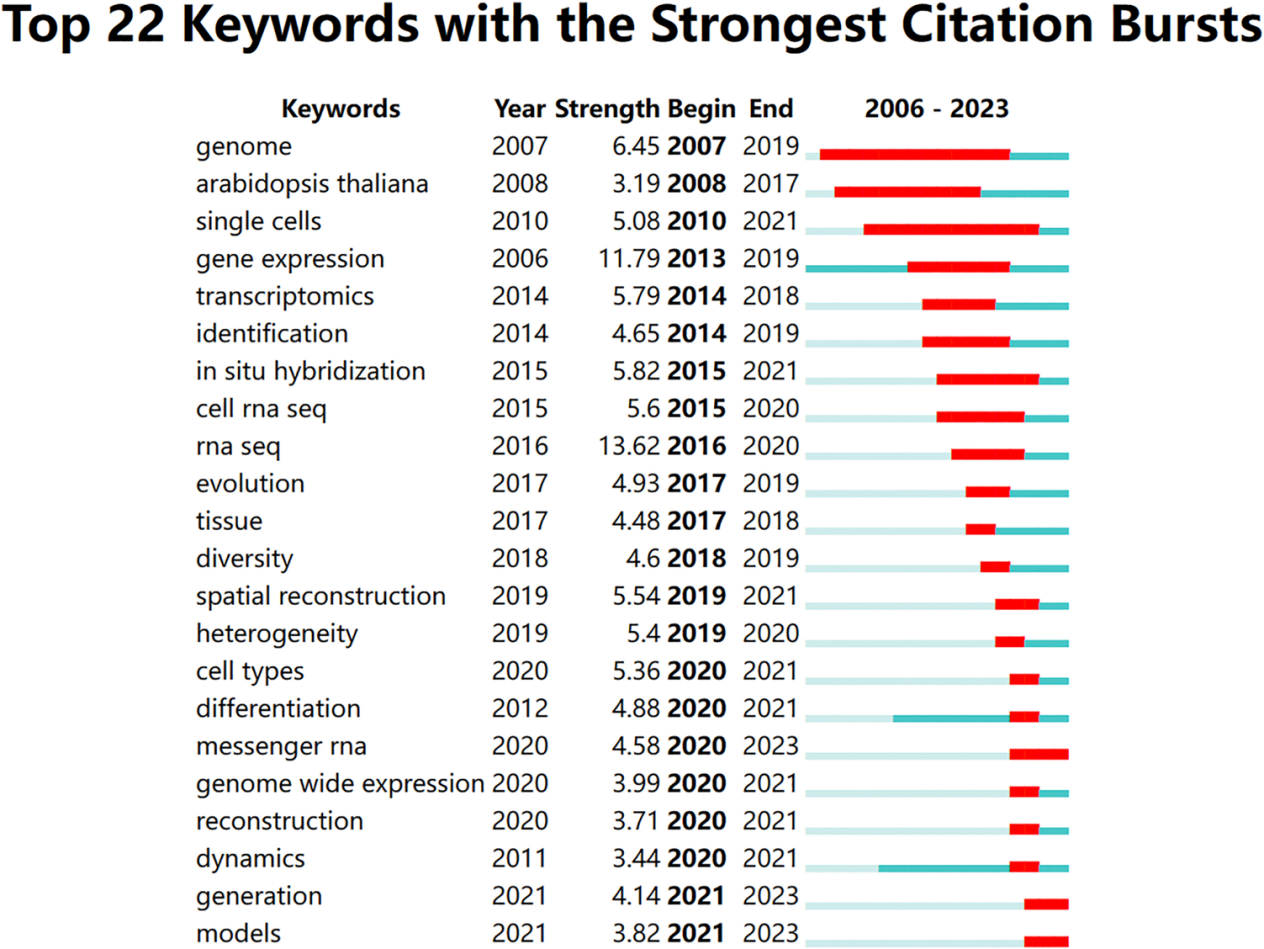
Top 22 keywords with the strongest citation burst

Cluster analysis is a statistical method to categorize data based on the degree of similarity, with the aim in this study of discovering the distribution of research content on a specific topic (Ai et al. 2022). We clustered the keywords using CiteSpace software (Fig. 6C). Seventeen clusters were extracted and labeled with “#” in the clustering diagram (Fig. 7C shows the first seven clusters). Cluster #0 labeled with “spatial transcriptomics” contains 10 cooccurring keywords: spatial transcriptomics; single-cell rna sequencing; precision medicine; synaptic plasticity; protein; gene expression; tissue; rna-seq; resolution; spatial reconstruction. The #1 macrophages cluster includes 10 keywords: multiomics analyses, single-cell omics, systems biology, to-cell interactions, and immune checkpoint inhibitors. Cluster #2 is related to cells and includes 10 keywords: gene expression; architecture; atlas; tissue; 4 thio 2 deoxyuridine; spatial transcriptomics; single-cell technology; molecular barcode; bulb; class i. Cluster #3 focuses on the topic of spatial expression, with 10 keywords in the cluster, including transcription factors; spatial expression; root meristem; abiotic stress; arabidopsis lyrata; arabidopsis lyrata; arabidopsis halleri; transcription factors; spatial expression; root meristem.

In Fig. 6B, we also visualize the average year of occurrence with different colors. Purple indicates previous keywords, and yellow indicates recent keywords. The keyword timeline view shows the evolution of high-frequency keywords, suggesting that the research hotspots in this field have gradually shifted from technology-related topics in the early stage to cancer, tumor, and immunity.

Burst detection identifies sudden increases in frequency over a short period of time (Fig. 7), revealing research hotspots over time and reflecting the evolutionary trend of hotspots. Figure 7 shows the top 22 keywords with citation bursts. Research hotspots in ST shifted from “cell rna seq” in 2015 to “generation” and “models” in 2021.

## Discussion

### General Information

Since the 1st ST-related publication in 2006, the number of annual publications has been in the single digits for the following 9 years. As of 2019, the percentage of ST publications among digital research literature remains low. 2020 marks a turning point for the discipline, as more researchers are focusing on ST and publishing articles on it, with a rapid upward trend.

Based on the analysis of cooperation between countries, institutions, and authors, we can identify some trends regarding the development of ST. First, there is an increasing number of countries involved in this field of research on a global scale. However, most of the published works come from a small number of countries and organizations. In the last 10 years, the top 10 countries accounted for more than 90% of all publications, while the top 10 institutions accounted for more than 30% of all publications, and of the top 10 organizations, four are from the United States and two are from Sweden, implying an imbalance in the leadership of these countries and organizations and in the academic resources in ST research. It is noteworthy that China is the only developing country among the top 10 most productive countries and ranks second in terms of production. Among the top 10 countries, the United States, Sweden, China, and the United Kingdom play a “bridging” role in ST research, especially Sweden, which ranks second in terms of publications but has a higher number of citations per study, significantly stronger links with other countries on the collaboration map, and more top researchers with frequently cited articles, suggesting Sweden's focus on innovation and collaboration in ST.

The results of bibliometric studies are based on bibliographic data from published articles and reviews. Therefore, it is important to determine where these studies are published, as the core sources provide the foundation and important evidence for the research field and influence future research directions (Deng et al. 2020). The results show that Nature Communications publishes the most ST-related literature and has been more active in recent years, providing an effective publishing platform for scholarly communication. On the other hand, the most cited journals play a key role in linking and informing subsequent studies (Brandt et al. 2019). Our findings show that the top 10 cited journals and the journals with the most cited references are world-renowned, high-quality journals, which confirms the importance and innovation of ST techniques. Nature is the most popular co-cited journal with the second highest IF among the top 10 most productive journals. It and its sub-journals accounted for 40% of the top 10 journals in the published literature, suggesting that they have a stronger focus on the ST field.

Co-citation analysis is a means of revealing patterns inherent in a category of research literature over time, and highly co-cited studies are often considered the foundation of research in a field (Lijun et al. 2019), do not reflect the knowledge base of the field, and provide guidance and evidence for future research.

In 2016, Karolinska institute researcher Patrik L. Ståhl published the most co-cited study in ST in collaboration with 20 other researchers (Linnarsson et al. 2016). The article describes a new strategy called “spatial transcriptomics” that enables spatially resolved visualization and quantitative analysis of the transcriptome in individual tissue sections. The authors demonstrate the feasibility and efficiency of spatial transcriptomics using mouse brain and human breast cancer tissue sections, demonstrate the feasibility of generating cDNAs from mRNAs in surface tissue sections, and show that spatial transcriptomics can provide quantitative gene expression data and visualization of mRNA distribution in tissue sections. This technique promises to enable novel bioinformatics analyses with applications in research and diagnostics.

Samuel G. Rodriques published an article in the Experimental category in 2019 that was the second most co-cited article (Rodriques et al. 2019). A new method called “Slide-seq” is described for obtaining high-resolution spatially resolved gene expression data. The researchers developed a technique to transfer RNA from tissue sections onto surfaces covered with DNA bar beads so that the location of the RNA can be inferred by sequencing. They demonstrated the effectiveness of Slide-seq by localizing cell types in the cerebellum and hippocampus, describing spatial gene expression patterns in the mouse cerebellum, and investigating cell type-specific responses in a mouse model of traumatic brain injury. Slide-seq can be applied to a range of tissues and provides high spatial resolution for detecting fine spatial features, providing a powerful tool for investigating gene expression in complex tissues at high spatial resolution. Additional spatial gene expression patterns were also revealed, such as genes that are exclusively expressed in or excluded from specific regions of the cerebellum. Overall, the development and application of Slide-seq as a scalable high-resolution method for measuring genome-wide gene expression in complex tissues is highlighted.

### Research Hotspots

Keywords are essential in hotspot analysis, and basic concepts such as “gene expression,” “expression,” and “spatial transcriptomics” ranked in the top 3 (Table 8), while “tissue” and “identification” ranked in the sixth and seventh places, illustrating the characteristics and features of ST. One of the main drivers of the rapid development of ST is that tissue context contributes to the assessment of cell biology (Germain et al. 2012).

Diseases are characterized by abnormal spatial organization within tissues, and ST techniques can improve our understanding of tissue structure and its molecular basis in health and disease. At a finer level, ST can reveal tissue neighborhoods and local features that contribute to disease.

Since ST techniques provide an unbiased map of spatial composition, they are used to generate tissue atlases that provide a valuable resource as reference maps. The brain is a good example of the most important and complex organ in mammals. Spatial heterogeneity is crucial, as tissues in different regions of the brain have different functions and cell types. In neuroscience, the advantages of ST are twofold. First, it eliminates the need to separate fragile neuronal tissues. Second, it preserves the spatial context of the cell (Williams et al. 2022). This means that gene expression in thousands of cells can be assessed in the context of structural organization, and such a map of the brain will hopefully ultimately reveal the spatial heterogeneity of this complex organ, which is essential for gaining insight into the path mechanisms of brain diseases. In the case of Alzheimer's disease, for example, ST techniques can provide a better understanding of the molecular mechanisms of AD neuropathology in a spatial context. Some researchers have analyzed the transcriptome profiles of AD and control MTG samples using the 10 × Visium platform, and they have identified unique marker genes in the cortical layer and white matter in AD compared to controls, as well as layer-specific differentially expressed genes (Chen et al. 2021). These are findings that can help to gain insights into the complex structure of the MTG and cellular responses to AD pathology.

*Regarding the application of ST in cancer and its contribution to immunotherapy*. The WOS autocategorization of research directions showed that oncology was the medical field with the most ST publications, while the keyword analysis results showed that “cancer,” “heterogeneity,” and “immunotherapy” appeared more frequently, located in the 12th, 13th, and 31th positions, respectively, and “tumor microenvironment” was constantly mentioned in some cutting-edge studies (T-cell dysfunction in the glioblastoma microenvironment is mediated by myeloid cells releasing interleukin-10; Longo et al. 2021; Wu et al. 2020).

In recent years, ST technology has developed rapidly and has been widely used in the construction of spatial organization maps and tumor heterogeneity research. To date, ST has been used to analyze the spatial heterogeneity of multiple cancer types. In addition, ST also helps to identify and provide a more comprehensive understanding of special spatial regions, such as tumor interfaces and tertiary lymphoid structures (TLSs), with unique tumor microenvironments (TMEs). Moreover, the construction of the ST atlas has improved the understanding of the TME, and the study of the ST combined with mass spectrometry or high-dimensional immunofluorescence methods will help in exploring the extent and origin of tumor heterogeneity and in obtaining information for targeted diagnosis and therapy.

In clinical practice, cancer treatment mainly includes surgery, radiotherapy, chemotherapy, targeted therapy, and immunotherapy. However, due to the existence of drug resistance in various cancer therapies, treatment failure occasionally occurs. The molecular heterogeneity of cancer is one of the main reasons for treatment failure related to drug resistance (Dagogo-Jack and Shaw 2018). Therefore, a better understanding of cancer heterogeneity will contribute to more precise diagnosis and improved patient prognosis. In this regard, ST holds great promise in deciphering complex cancer heterogeneity by providing gene expression information indexed to loci.

Malignant tumors have considerable heterogeneity, including intertumor heterogeneity and intratumor heterogeneity (Wu et al. 2021). Tumor heterogeneity is the result of a combination of internal and external factors. External factors are mainly related to the TME (Bao et al. 2021). The TME mainly consists of tumor cells, immune cells, and stromal cells, which are important mediators of cancer progression and therapeutic outcomes. The TME plays a crucial role in tumorigenesis, growth, invasion, and metastasis and has multiple ways of inducing both beneficial and undesirable outcomes (Jin and Jin 2020; Quail and Joyce 2013). TME analysis helps in identifying potential therapeutic targets.

The composition, proportion, location, and motility of interdependent cells in the TME have a significant impact on the outcome of cancer immunotherapy (Pinato et al. 2020). These factors have a major impact on cancer progression and response to treatment and are one of the major determinants of patient prognosis (Lei et al. 2020). Therefore, visualizing antitumor cells in the TME and exploring their interactions may help, by better assessing tumor heterogeneity, in identifying patients who could benefit from treatment. Thus, to improve the efficacy of immunotherapy, ST technology can be utilized to identify the source of tumor heterogeneity and provide potential therapeutic targets for treatment.

One study identified a specific spatial structure in some hepatocellular carcinoma cells by combining ST and scRNA-seq and termed it the tumor immune barrier (TIB). It is formed by the interaction of SPP1 + macrophages and CAFs from immunotherapy-naïve patients, and this structure limits the infiltration of immune cells into the tumor core. In vitro, SPP1 expression was upregulated in macrophages under hypoxic conditions. This result suggests that disruption of SPP1 + macrophage interactions with malignant hepatocytes and CAFs in combination with anti-PD-1 therapy may improve efficacy (Liu et al. 2023). This provides a novel therapeutic strategy to treat tumors by disrupting cell‒cell interactions and combining them with immune checkpoint inhibitors.

Glioblastoma is highly resistant due to its TME, which disables antitumor immune responses. Some researchers have identified a subpopulation of IL-10-releasing HMOX1 + myeloid cells that spatially localize to mesenchymal-like tumor regions, driving T-cell exhaustion and contributing to the immunosuppressive tumor microenvironment, as revealed by ST technology and other methods of integration. T-cell function was found to be rescued by inhibiting the JAK/STAT pathway, demonstrating that IL-10 release is an important driver of tumor immune escape (Ravi et al. 2022). These findings contribute to the development of successful immunotherapies and provide new ideas for the treatment of various types of tumors.

Keyword Bursts refers to a sudden increase in the frequency of a research topic within a short period of time, revealing a research hotspot over a period of time and reflecting the evolutionary trend of the hotspot. “Differentiation” has been hotly researched in recent years, and “models,” which appeared in 2021 and continues to this day, is the latest explosion of words, suggesting that they may be a research hotspot in the coming years.

*An overview of the role of Big Data Intelligence Models in the development of ST technologies*. With the emergence of new ST technologies, ST analysis faces many new computational challenges. Since the spatial context of tissues is highly correlated with gene expression, cell type distribution, cell‒cell communication, and cell function, new computational approaches are needed to analyze ST data, while making full use of the added spatial information. To address the challenges of computing ST, many new computational methods have been developed, of which deep learning based on neural networks is the main type. These methods have been developed to address different aspects of ST analysis, including the detection of spatially variable genes, clustering analysis of spots or genes, communication analysis, cell type deconvolution, and enhanced spatial gene expression (Li et al. 2022b).

Deep learning models is a type of machine learning that utilizes deep artificial neural networks, often characterized by algorithms consisting of multiple layers of artificial neural networks. The earliest application of deep learning for image recognition, reported in a 2014 paper by Dong et al. (2015), first proposed the use of deep convolutional neural networks to learn the end-to-end mapping relationship between low-resolution images and high-resolution images in the task of image recognition. Recent advances in deep learning have shown that the diagnosis of various diseases based on the classification of radiographic images and tissue slices is almost beyond human capabilities (Teare et al. 2017; Veta et al. 2015).

Compared to other methods, deep learning performance excels in cancer analysis, which is crucial for improving precision oncology. The deep learning technique can overcome data complexity and reveal cancer heterogeneity, while reducing time and cost. Specifically, deep learning models can connect spatial and single-cell data to achieve super resolution of tumor specimens, including cell‒cell interactions and cell-type annotations. These models reduce experimental costs while accomplishing feature extraction, helping pathologists and cancer researchers (Liu et al. 2019; Qian et al. 2020; Xiong et al. 2019; Halawani et al. 2023). In addition, the method can be used individually for each data type to study tumor heterogeneity, enabling its limitations and challenges to be overcome.

The essence of differentiation is the selective expression of the genome in time and space, and ST has greatly improved our understanding of this in space. Stem cells are the most differentiated cells after embryonic maturation, and ST helps to identify the molecular signals and factors that regulate stem cell development and differentiation, which will help us to conduct more in-depth studies on human growth and development. Recent advances in ST have greatly improved our understanding of stem cell biology, and by combining ST with other techniques such as single-cell transcriptomics and population-level gene expression profiling, researchers can gain a comprehensive view of the molecular landscape in stem cell niches. These advances have led to the identification of secreted factors that promote human hematopoietic stem cell (HSC) development. For example, a study by Crosse et al. (2020) used multilayer ST to analyze signaling in the embryonic niche of human hematopoietic stem cells. They identified secreted proteins such as endothelin, which are located proximal to the site of hematopoietic stem cell emergence and demonstrated their role in promoting hematopoiesis in mice and humans. Advances in ST have provided valuable insights into cellular relationships and heterogeneity within stem cell populations. This technique is particularly useful for studying bone marrow stromal cells and their interactions with hematopoietic stem cells (HSCs). There have been studies that have identified new subpopulations and transcriptional heterogeneity in what were previously thought to be homogeneous compartments by ST techniques, including the discovery of LeprþCxcl12-enriched reticulocytes as a major source of pro-hematopoietic factors, and there have also been studies that have clarified the molecular characterization and localization of bone marrow-resident cell types(Al-Sabah et al. 2019). In addition, differentiation and development in the heart, brain, endocrine, and other tissues are continuing to deepen with ST technologies (Fan et al. 2023; Luo et al. 2021; Mantri et al. 2021; Matsumoto and Yamamoto 2024; Zhong et al. 2023), improving our knowledge of human development and related diseases. In conclusion, ST is a valuable tool for the study of stem cell biology and can provide important insights into the molecular mechanisms of stem cell development and differentiation.

## Conclusion

In conclusion, in this study, the bibliometric analysis of ST over the period 2006–2023 has been conducted through automated analysis software. We found that there is a growing interest in this field worldwide, with the United States being the leading country with the most publications and China being one of the most active major players, but collaboration and communication among countries and institutions needs to be strengthened. The cutting-edge directions and hotspots in this field are tissue recognition, cancer, heterogeneity, immunotherapy, differentiation, and artificial intelligence model (Covert et al. 2023; Li et al. 2022a, 2022b; Liu et al. 2023; Williams et al. 2022; Zhong et al. 2023). Updates in ST technology have provided higher precision and accuracy for tissue recognition, allowing a more comprehensive study of tissues at the molecular level. The application of ST has gradually spread from cancer and immunotherapy to other disease areas under continuous development. In the future, the combination of ST technology with big data, artificial intelligence, and other technologies will continue to optimize its analytical capabilities to better serve the cause of human health. We expect more advanced and effective ST technologies to enter clinical applications soon and bring hope to patients.

## Supplementary Information

Below is the link to the electronic supplementary material.

Supplementary file1 (DOCX 241 kb)

## Data Availability

The datasets presented in this study can be found in online repositories. The names of the repository/repositories and accession number(s) can be found in the article/Supplementary Material.

## References

[CR1] Ai Y, Xing Y, Yan L, Ma D, Gao A, Xu Q, Zhang S, Mao T, Pan Q, Ma X, Zhang J (2022) Atrial Fibrillation and Depression: A Bibliometric Analysis From 2001 to 2021. Front Cardiovasc Med 9:775329. 10.3389/fcvm.2022.775329 35252380 10.3389/fcvm.2022.775329 PMC8888833

[CR2] Al-Sabah J, Baccin C, Haas S (2019) Single-cell and spatial transcriptomics approaches of the bone marrow microenvironment. Curr Opin Oncol 32(2):146–153 10.1097/CCO.0000000000000602 31833957

[CR3] Aria M, Cuccurullo C (2017) bibliometrix: an R-tool for comprehensive science mapping analysis. J Informet 11(4):959–975. 10.1016/j.joi.2017.08.007

[CR4] Bao Z, Wang Y, Wang Q, Fang S, Shan X, Wang J, Jiang T (2021) Intratumor heterogeneity, microenvironment, and mechanisms of drug resistance in glioma recurrence and evolution. Front Med 15(4):551–561. 10.1007/s11684-020-0760-2 33893983 10.1007/s11684-020-0760-2

[CR5] Brandt JS, Hadaya O, Schuster M, Rosen T, Ananth CV (2019) A bibliometric analysis of top-cited journal articles in obstetrics and gynecology. JAMA Netw Open 2(12):e1918007 31860106 10.1001/jamanetworkopen.2019.18007 PMC6991228

[CR6] Chen C (2004) Searching for intellectual turning points: progressive knowledge domain visualization. Proc Natl Acad Sci USA 101(Suppl 1):5303–5310. 10.1073/pnas.0307513100 14724295 10.1073/pnas.0307513100 PMC387312

[CR7] Chen C, Hu Z, Liu S, Tseng H (2012) Emerging trends in regenerative medicine: a scientometric analysis in CiteSpace. Expert Opin Biol Ther 12(5):593–608. 10.1517/14712598.2012.674507 22443895 10.1517/14712598.2012.674507

[CR8] Chen S, Chang Y, Li L, Acosta D, Morrison C, Wang C, Julian D, Hester ME, Serrano G, Beach T (2021) Spatially resolved transcriptomics reveals gene signatures underlying the vulnerability of human middle temporal gyrus in Alzheimer's disease. SSRN Electron J 10.1186/s40478-022-01494-6 PMC9773466 36544231

[CR9] Covert I, Gala R, Wang T, Svoboda K, Sumbul U, Lee SI (2023) Predictive and robust gene selection for spatial transcriptomics. Nat Commun 14(1):2091. 10.1038/s41467-023-37392-1 37045821 10.1038/s41467-023-37392-1 PMC10097645

[CR10] Crosse EI, Gordon-Keylock S, Rybtsov S, Binagui-Casas A, Felchle H, Nnadi NC, Kirschner K, Chandra T, Tamagno S, Webb DJ (2020) Multi-layered spatial transcriptomics identify secretory factors promoting human hematopoietic. Stem Cell Dev 27:822–839 10.1016/j.stem.2020.08.004 PMC7671940 32946788

[CR11] Dagogo-Jack I, Shaw AT (2018) Tumour heterogeneity and resistance to cancer therapies. Nat Rev Clin Oncol 15(2):81–94. 10.1038/nrclinonc.2017.166 29115304 10.1038/nrclinonc.2017.166

[CR12] Deng Z, Wang H, Chen Z, Wang T (2020) Bibliometric analysis of dendritic epidermal T Cell (DETC) research from 1983 to 2019. Front Immunol 11:259 32226424 10.3389/fimmu.2020.00259 PMC7080701

[CR13] Dong C, Loy CC, He K, Tang X (2015) Image super-resolution using deep convolutional networks. IEEE Trans Pattern Anal Mach Intell 38:295–307 10.1109/TPAMI.2015.2439281 26761735

[CR14] Ecker JR, Geschwind DH, Kriegstein AR, Ngai J, Osten P, Polioudakis D, Regev A, Sestan N, Wickersham IR, Zeng H (2017) The BRAIN initiative cell census consortium: lessons learned toward generating a comprehensive brain cell atlas. Neuron 96(3):542–557. 10.1016/j.neuron.2017.10.007 29096072 10.1016/j.neuron.2017.10.007 PMC5689454

[CR15] Fan Z, Luo Y, Lu H, Wang T, Feng Y, Zhao W, Kim P, Zhou X (2023) SPASCER: spatial transcriptomics annotation at single-cell resolution. Nucleic Acids Res 51(D1):D1138–D1149. 10.1093/nar/gkac889 36243975 10.1093/nar/gkac889 PMC9825565

[CR16] Ge Y, Chao T, Sun J, Liu W, Chen Y, Wang C (2022) Frontiers and hotspots evolution in psycho-cardiology: a bibliometric analysis from 2004 to 2022. Curr Probl Cardiol 47(12):101361. 10.1016/j.cpcardiol.2022.101361 35995242 10.1016/j.cpcardiol.2022.101361

[CR17] Germain RN, Robey EA, Cahalan MD (2012) A decade of imaging cellular motility and interaction dynamics in the immune system. Science 336:1676–1681 22745423 10.1126/science.1221063 PMC3405774

[CR18] Giacomello S (2021) A new era for plant science: spatial single-cell transcriptomics. Curr Opin Plant Biol 60:102041. 10.1016/j.pbi.2021.102041 33915520 10.1016/j.pbi.2021.102041

[CR19] Guler AT, Waaijer CJF, Palmblad M (2016) Scientific workflows for bibliometrics. Scientometrics 107(2):385–398 27122644 10.1007/s11192-016-1885-6 PMC4833826

[CR20] Halawani R, Buchert M, Chen YP (2023) Deep learning exploration of single-cell and spatially resolved cancer transcriptomics to unravel tumour heterogeneity. Comput Biol Med 164:107274. 10.1016/j.compbiomed.2023.107274 37506451 10.1016/j.compbiomed.2023.107274

[CR21] Hassan-Montero Y, De-Moya-Anegón F, Guerrero-Bote VP (2022) SCImago Graphica: a new tool for exploring and visually communicating data. El Profesional De La Información. 10.3145/epi.2022.sep.02

[CR22] Jin MZ, Jin WL (2020) The updated landscape of tumor microenvironment and drug repurposing. Signal Transduct Target Ther 5(1):166. 10.1038/s41392-020-00280-x 32843638 10.1038/s41392-020-00280-x PMC7447642

[CR23] Lara AR, Leal L, Flores N, Gosset G, Bolívar F, Ramírez OT (2006) Transcriptional and metabolic response of recombinant *Escherichia coli* to spatial dissolved oxygen tension gradients simulated in a scale-down system. Biotechnol Bioeng 92:372–385 10.1002/bit.20704 16187334

[CR24] Lei X, Lei Y, Li JK, Du WX, Li RG, Yang J, Li J, Li F, Tan HB (2020) Immune cells within the tumor microenvironment: biological functions and roles in cancer immunotherapy. Cancer Lett 470:126–133. 10.1016/j.canlet.2019.11.009 31730903 10.1016/j.canlet.2019.11.009

[CR25] Li Q, Zhang X, Ke R (2022a) Spatial transcriptomics for tumor heterogeneity analysis. Front Genet 13:906158. 10.3389/fgene.2022.906158 35899203 10.3389/fgene.2022.906158 PMC9309247

[CR26] Li Y, Stanojevic S, Garmire LX (2022b) Emerging artificial intelligence applications in spatial transcriptomics analysis. Comput Struct Biotechnol J 20:2895–2908. 10.1016/j.csbj.2022.05.056 35765645 10.1016/j.csbj.2022.05.056 PMC9201012

[CR27] Lijun Y, Liangxiu H, Naxin L (2019) A new approach to journal co-citation matrix construction based on the number of co-cited articles in journals. Scientometrics 120(2):507–517

[CR28] Linnarsson S, Codeluppi S, Vickovic S, Huss M, Mulder S (2016) Visualization and analysis of gene expression in tissue sections by spatial transcriptomics. Science 353(6294):78–82 27365449 10.1126/science.aaf2403

[CR29] Liu Q, Lv H, Jiang R (2019) hicGAN infers super resolution Hi-C data with generative adversarial networks. Bioinformatics 35(14):i99–i107 31510693 10.1093/bioinformatics/btz317 PMC6612845

[CR30] Liu Y, Xun Z, Ma K, Liang S, Li X, Zhou S, Sun L, Liu Y, Du Y, Guo X, Cui T, Zhou H, Wang J, Yin D, Song R, Zhang S, Cai W, Meng F, Guo H, Zhang B, Yang D, Bao R, Hu Q, Wang J, Ye Y, Liu L (2023) Identification of a tumour immune barrier in the HCC microenvironment that determines the efficacy of immunotherapy. J Hepatol 78(4):770–782. 10.1016/j.jhep.2023.01.011 36708811 10.1016/j.jhep.2023.01.011

[CR31] Longo SK, Guo MG, Ji AL, Khavari PA (2021) Integrating single-cell and spatial transcriptomics to elucidate intercellular tissue dynamics. Nat Rev Genet 22(10):627–644. 10.1038/s41576-021-00370-8 34145435 10.1038/s41576-021-00370-8 PMC9888017

[CR32] Luo W, Lin GN, Song W, Zhang Y, Lai H, Zhang M, Miao J, Cheng X, Wang Y, Li W, Wei W, Gao WQ, Yang R, Wang J (2021) Single-cell spatial transcriptomic analysis reveals common and divergent features of developing postnatal granule cerebellar cells and medulloblastoma. BMC Biol 19(1):135. 10.1186/s12915-021-01071-8 34210306 10.1186/s12915-021-01071-8 PMC8247169

[CR33] Mantri M, Scuderi GJ, Abedini-Nassab R, Wang MFZ, McKellar D, Shi H, Grodner B, Butcher JT, De Vlaminck I (2021) Spatiotemporal single-cell RNA sequencing of developing chicken hearts identifies interplay between cellular differentiation and morphogenesis. Nat Commun 12(1):1771. 10.1038/s41467-021-21892-z 33741943 10.1038/s41467-021-21892-z PMC7979764

[CR34] Marx V (2021) Method of the year: spatially resolved transcriptomics. Nat Methods 18(1):9–14. 10.1038/s41592-020-01033-y 33408395 10.1038/s41592-020-01033-y

[CR35] Matsumoto R, Yamamoto T (2024) Single-cell and spatial transcriptomics in endocrine research. Endocr J 71(2):101–118. 10.1507/endocrj.EJ23-0457 38220200 10.1507/endocrj.EJ23-0457

[CR36] Meng T, Wang P, Ding J, Du R, Gao J, Li A, Yu S, Liu J, Lu X, He Q (2022) Global research trends on ventricular remodeling: a bibliometric analysis from 2012 to 2022. Curr Probl Cardiol 47(11):101332. 10.1016/j.cpcardiol.2022.101332 35870550 10.1016/j.cpcardiol.2022.101332

[CR37] Mortazavi A, Williams BA, McCue K, Schaeffer L, Wold B (2008) Mapping and quantifying mammalian transcriptomes by RNA-Seq. Nat Methods 5(7):621–628. 10.1038/nmeth.1226 18516045 10.1038/nmeth.1226 PMC13303166

[CR38] Ozato Y, Kojima Y, Kobayashi Y, Hisamatsu Y, Toshima T, Yonemura Y, Masuda T, Kagawa K, Goto Y, Utou M, Fukunaga M, Gamachi A, Imamura K, Kuze Y, Zenkoh J, Suzuki A, Niida A, Hirose H, Hayashi S, Koseki J, Oki E, Fukuchi S, Murakami K, Tobo T, Nagayama S, Uemura M, Sakamoto T, Oshima M, Doki Y, Eguchi H, Mori M, Iwasaki T, Oda Y, Shibata T, Suzuki Y, Shimamura T, Mimori K (2023) Spatial and single-cell transcriptomics decipher the cellular environment containing HLA-G+ cancer cells and SPP1+ macrophages in colorectal cancer. Cell Rep 42(1):111929. 10.1016/j.celrep.2022.111929 36656712 10.1016/j.celrep.2022.111929

[CR39] Park HE, Jo SH, Lee RH, Macks CP, Ku T, Park J, Lee CW, Hur JK, Sohn CH (2023) Spatial transcriptomics: technical aspects of recent developments and their applications in neuroscience and cancer research. Adv Sci (weinh) 10(16):e2206939. 10.1002/advs.202206939 37026425 10.1002/advs.202206939 PMC10238226

[CR40] Pinato DJ, Guerra N, Fessas P, Murphy R, Mineo T, Mauri FA, Mukherjee SK, Thursz M, Wong CN, Sharma R, Rimassa L (2020) Immune-based therapies for hepatocellular carcinoma. Oncogene 39(18):3620–3637. 10.1038/s41388-020-1249-9 32157213 10.1038/s41388-020-1249-9 PMC7190571

[CR41] Qian X, Harris KD, Hauling T, Nicoloutsopoulos D, Nilsson M (2020) Probabilistic cell typing enables fine mapping of closely related cell types in situ. Nat Methods 17(1):1–6 31740815 10.1038/s41592-019-0631-4 PMC6949128

[CR42] Quail DF, Joyce JA (2013) Microenvironmental regulation of tumor progression and metastasis. Nat Med 19(11):1423–1437. 10.1038/nm.3394 24202395 10.1038/nm.3394 PMC3954707

[CR43] Rao A, Barkley D, França GS, Yanai I (2021) Exploring tissue architecture using spatial transcriptomics. Nature 596(7871):211–220. 10.1038/s41586-021-03634-9 34381231 10.1038/s41586-021-03634-9 PMC8475179

[CR44] Ravi VM, Neidert N, Will P, Joseph K, Maier JP, Kückelhaus J, Vollmer L, Goeldner JM, Behringer SP, Scherer F, Boerries M, Follo M, Weiss T, Delev D, Kernbach J, Franco P, Schallner N, Dierks C, Carro MS, Hofmann UG, Fung C, Sankowski R, Prinz M, Beck J, Salié H, Bengsch B, Schnell O, Heiland DH (2022) T-cell dysfunction in the glioblastoma microenvironment is mediated by myeloid cells releasing interleukin-10. Nat Commun 13(1):925. 10.1038/s41467-022-28523-1 35177622 10.1038/s41467-022-28523-1 PMC8854421

[CR45] Regev A, Teichmann SA, Lander ES, Amit I, Benoist C, Birney E, Bodenmiller B, Campbell P, Carninci P, Clatworthy M, Clevers H, Deplancke B, Dunham I, Eberwine J, Eils R, Enard W, Farmer A, Fugger L, Göttgens B, Hacohen N, Haniffa M, Hemberg M, Kim S, Klenerman P, Kriegstein A, Lein E, Linnarsson S, Lundberg E, Lundeberg J, Majumder P, Marioni JC, Merad M, Mhlanga M, Nawijn M, Netea M, Nolan G, Pe’er D, Phillipakis A, Ponting CP, Quake S, Reik W, Rozenblatt-Rosen O, Sanes J, Satija R, Schumacher TN, Shalek A, Shapiro E, Sharma P, Shin JW, Stegle O, Stratton M, Stubbington MJT, Theis FJ, Uhlen M, van Oudenaarden A, Wagner A, Watt F, Weissman J, Wold B, Xavier R, Yosef N (2017) The human cell atlas. Elife. 10.7554/eLife.27041 29206104 10.7554/eLife.27041 PMC5762154

[CR46] Rodriques SG, Stickels RR, Goeva A, Martin CA, Murray E, Vanderburg CR, Welch J, Chen LM, Chen F, Macosko EZ (2019) Slide-seq: a scalable technology for measuring genome-wide expression at high spatial resolution. Science 363(6434):1463–1467 30923225 10.1126/science.aaw1219 PMC6927209

[CR48] Teare P, Fishman M, Benzaquen O, Toledano E, Elnekave E (2017) Malignancy detection on mammography using dual deep convolutional neural networks and genetically discovered false color input enhancement. J Digit Imaging 30:499–505 28656455 10.1007/s10278-017-9993-2 PMC5537100

[CR49] Valberg SJ, Williams ZJ, Henry ML, Finno CJ (2023) Cerebellar axonopathy in Shivers horses identified by spatial transcriptomic and proteomic analyses. J Vet Intern Med 37(4):1568–1579. 10.1111/jvim.16784 37288990 10.1111/jvim.16784 PMC10365050

[CR50] van Eck NJ, Waltman L (2010) Software survey: VOSviewer, a computer program for bibliometric mapping. Scientometrics 84(2):523–538. 10.1007/s11192-009-0146-3 20585380 10.1007/s11192-009-0146-3 PMC2883932

[CR51] Veta M, Diest PJV, Willems SM, Wang H, Madabhushi A, Cruz-Roa A, Gonzalez F, Larsen ABL, Vestergaard JS, Dahl AB (2015) Assessment of algorithms for mitosis detection in breast cancer histopathology images. Med Image Anal 20:237–248 25547073 10.1016/j.media.2014.11.010

[CR52] Williams CG, Lee HJ, Asatsuma T, Vento-Tormo R, Haque A (2022) An introduction to spatial transcriptomics for biomedical research. Genome Med 14(1):68. 10.1186/s13073-022-01075-1 35761361 10.1186/s13073-022-01075-1 PMC9238181

[CR53] Wilson M, Sampson M, Barrowman N, Doja A (2021) Bibliometric analysis of neurology articles published in general medicine journals. JAMA Netw Open 4(4):e215840. 10.1001/jamanetworkopen.2021.5840 33856477 10.1001/jamanetworkopen.2021.5840 PMC8050738

[CR55] Wu K, Lin K, Li X, Yuan X, Xu P, Ni P, Xu D (2020) Redefining tumor-associated macrophage subpopulations and functions in the tumor microenvironment. Front Immunol 11:1731. 10.3389/fimmu.2020.01731 32849616 10.3389/fimmu.2020.01731 PMC7417513

[CR54] Wu F, Fan J, He Y, Xiong A, Yu J, Li Y, Zhang Y, Zhao W, Zhou F, Li W, Zhang J, Zhang X, Qiao M, Gao G, Chen S, Chen X, Li X, Hou L, Wu C, Su C, Ren S, Odenthal M, Buettner R, Fang N, Zhou C (2021) Single-cell profiling of tumor heterogeneity and the microenvironment in advanced non-small cell lung cancer. Nat Commun 12(1):2540. 10.1038/s41467-021-22801-0 33953163 10.1038/s41467-021-22801-0 PMC8100173

[CR56] Xavier-Santos D, Padilha M, Fabiano GA, Vinderola G, Gomes Cruz A, Sivieri K, Costa Antunes AE (2022) Evidences and perspectives of the use of probiotics, prebiotics, synbiotics, and postbiotics as adjuvants for prevention and treatment of COVID-19: a bibliometric analysis and systematic review. Trends Food Sci Technol 120:174–192. 10.1016/j.tifs.2021.12.033 35002079 10.1016/j.tifs.2021.12.033 PMC8720301

[CR57] Xiong L, Xu K, Tian K, Shao Y, Zhang QC (2019) SCALE method for single-cell ATAC-seq analysis via latent feature extraction. Nat Commun 10(1):4576 31594952 10.1038/s41467-019-12630-7 PMC6783552

[CR58] Xu X, Wang Y, Li Y, Zhang B, Song Q (2022a) The future landscape of macrophage research in cardiovascular disease: a bibliometric analysis. Curr Probl Cardiol 47(10):101311. 10.1016/j.cpcardiol.2022.101311 35810847 10.1016/j.cpcardiol.2022.101311

[CR59] Xu Y, Zhang Z, He J, Chen Z (2022b) Immune effects of macrophages in rheumatoid arthritis: a bibliometric analysis from 2000 to 2021. Front Immunol 13:903771. 10.3389/fimmu.2022.903771 36172378 10.3389/fimmu.2022.903771 PMC9510364

[CR60] Yang S, Zhao S, Ye Y, Jia L, Lou Y (2022) Global research trends on the links between gut microbiota and cancer immunotherapy: a bibliometric analysis (2012–2021). Front Immunol 13:952546. 10.3389/fimmu.2022.952546 36090978 10.3389/fimmu.2022.952546 PMC9449151

[CR61] Zhang X, Zhou Y, Wei N, Shou X, Fan S, You Y, Li Y, Hu Y (2022) A bibliometric analysis of heart failure with preserved ejection fraction from 2000 to 2021. Curr Probl Cardiol 47(9):101243. 10.1016/j.cpcardiol.2022.101243 35545178 10.1016/j.cpcardiol.2022.101243

[CR62] Zhong S, Wang M, Huang L, Chen Y, Ge Y, Zhang J, Shi Y, Dong H, Zhou X, Wang B, Lu T, Jing X, Lu Y, Zhang J, Wang X, Wu Q (2023) Single-cell epigenomics and spatiotemporal transcriptomics reveal human cerebellar development. Nat Commun 14(1):7613. 10.1038/s41467-023-43568-6 37993461 10.1038/s41467-023-43568-6 PMC10665552

