# Supplementary material for: A Bibliometric Analysis of the Spatial Transcriptomics Literature from 2006 to 2023
Source: Cell Mol Neurobiol. 2024 Jun 10;44:50. doi: 10.1007/s10571-024-01484-3 (PMC11164738; doi:10.1007/s10571-024-01484-3)
Supplement: Supplementary file 1 — Supplementary file1 (DOCX 241 kb) [file 10571_2024_1484_MOESM1_ESM.docx]

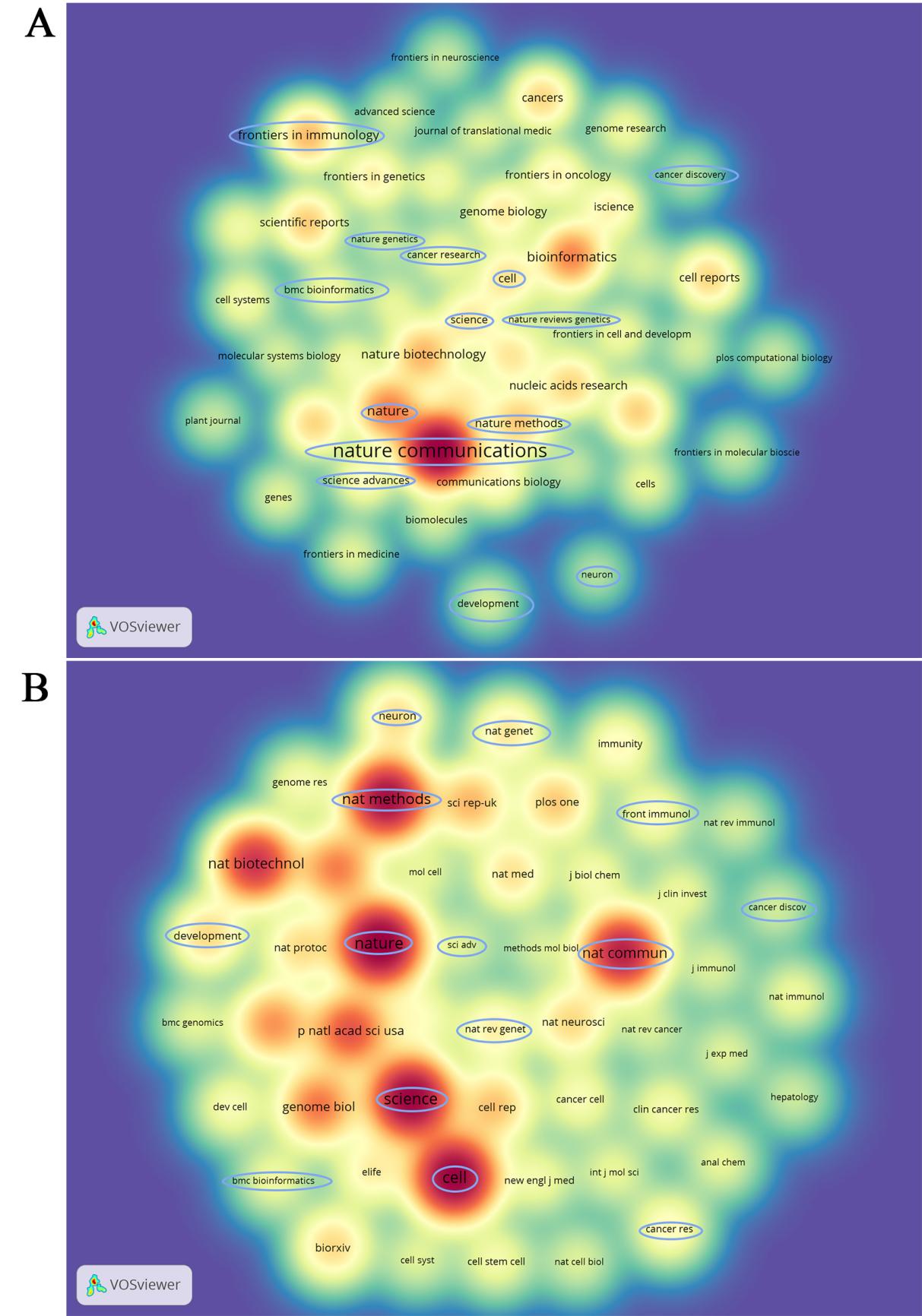


Supplemental FIGURE 1| The spectral density map of (A) citing and (B) co-cited journals. The blue oval circles mark the journals that are present in both panels.


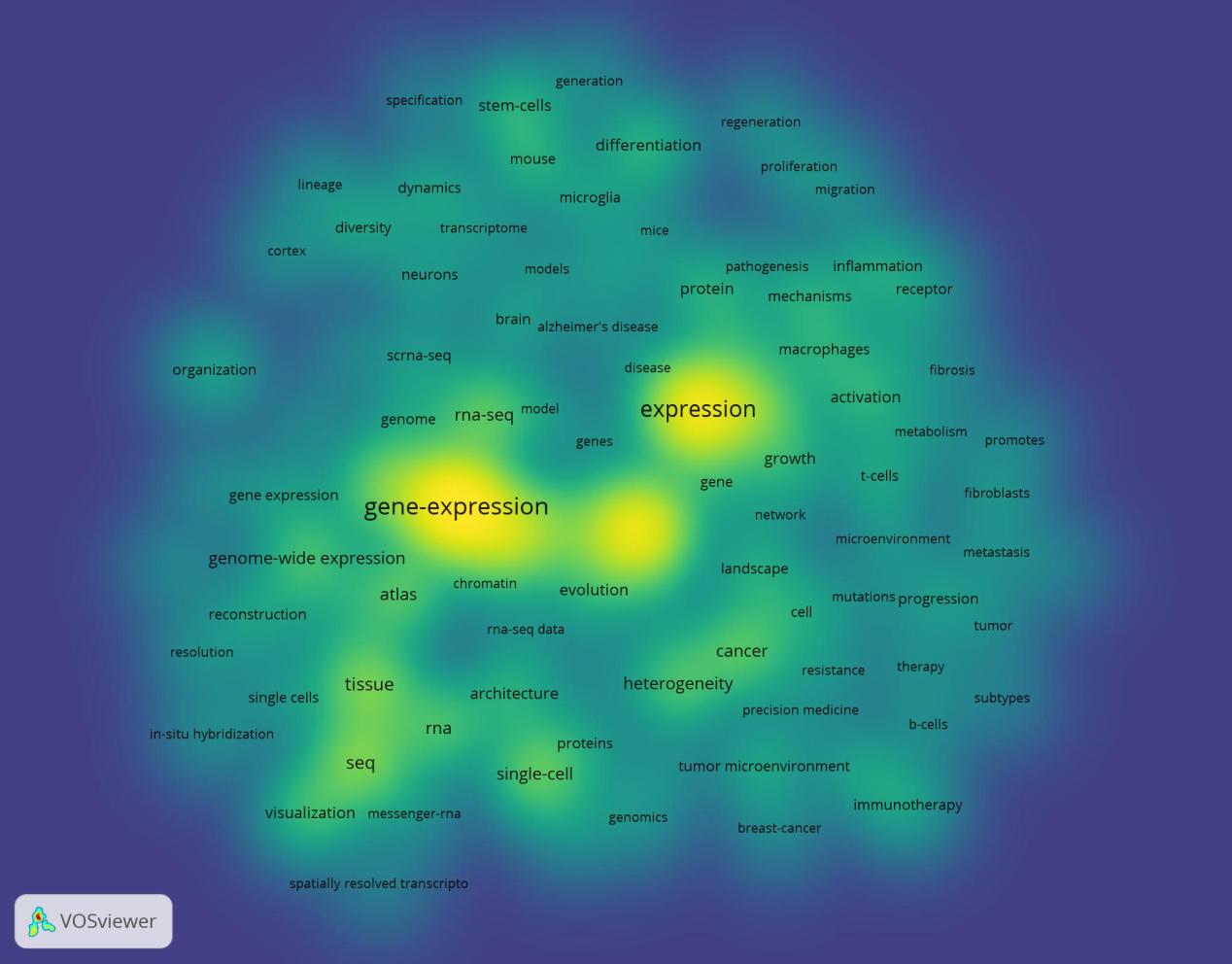


Supplemental FIGURE 2| The spectral density maps of the top 100 keywords.
